# High Levels of SIRT1 Expression as a Protective Mechanism Against Disease-Related Conditions

**DOI:** 10.3389/fendo.2018.00614

**Published:** 2018-10-15

**Authors:** Birsen Elibol, Ulkan Kilic

**Affiliations:** ^1^Department of Medical Biology, Faculty of Medicine, Bezmialem Vakif University, Istanbul, Turkey; ^2^Department of Medical Biology, Faculty of Medicine, University of Health Sciences, Istanbul, Turkey

**Keywords:** SIRT1 expression, oxidative stress, metabolic diseases, cardiovascular diseases, neurodegenerative diseases

## Abstract

SIRT1 protein, a member of Silent Information Regulator 2 (Sir2) protein family, have gained considerable attention as epigenetic regulators for a great area in the human physiology. Changes in sirtuin expression are critical in several diseases, including metabolic syndrome, cardiovascular diseases, cancer and neurodegeneration. Here, we provide an overview of the association of the increasing level of SIRT1 protein for regulating some disease related conditions such as obesity, cardiovascular diseases and neurodegeneration. This review also provides a detailed molecular understanding of the interaction of the some basic molecules with increasing SIRT1 levels rather than reduction of the SIRT1 expression. In this context, the current approaches to enhancing the expression of SIRT1 points the importance of epigenetics in several age-related diseases to provide a healthy aging by developing novel therapies which can prevent or damp the progression of some diseases.

## Introduction

Sirtuin 1 (SIRT1) which is encoded by the *SIRT1* gene is the most conserved mammalian nicotinamide adenine dinucleotide (NAD+) dependent histone deacetylase (1). Besides its role being a target for histone and non-histone proteins, SIRT1 functions as a transcription factor for many different physiological processes (2). According to the previous experiments which were performed using yeast, worms and flies as model organisms, sirtuins were accepted as evolutionarily conserved epigenetic mediators of longevity (3–5). In addition to the key role on extending life by regulating the response to some conditions such as fasting, caloric restriction and exercise, SIRT1 regulates many endocrine functions, protects organism from oxidative stress-related cellular events, promotes DNA stability, and decreases various age-related disorders, such as neurodegenerative disease, metabolic abnormalities, and cancer (6–9).

**Figure 1:**
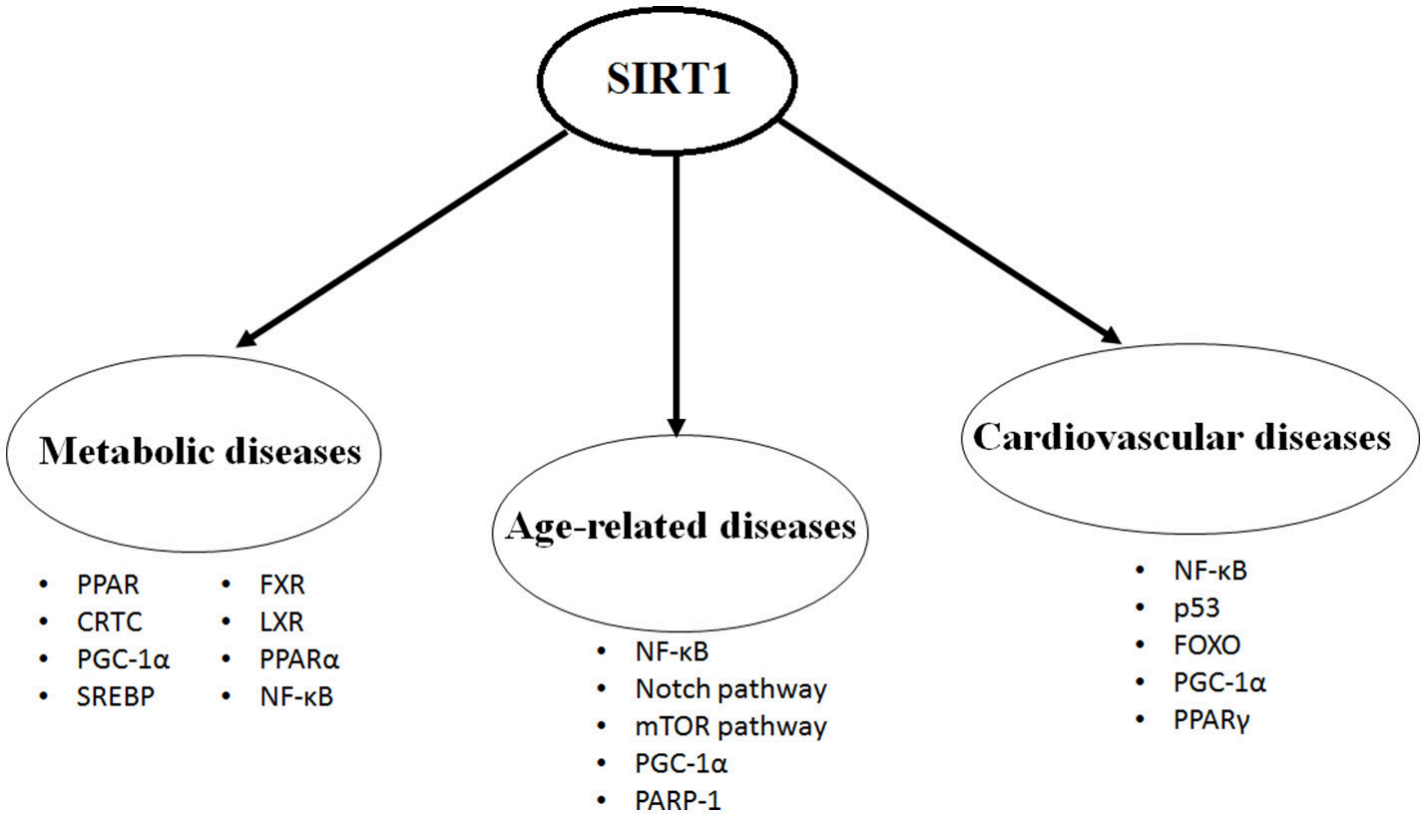
The most studied targets of SIRT1 in the metabolic diseases, age-related diseases and cardiovascular diseases.

SIRT1 protein is expressed in most of the body parts including brain, heart, kidney, liver, pancreas, spleen, skeletal muscle, endothelial tissue and white adipose tissue. By expression and activation of SIRT1, modulation of its downstream pathways occurs by targeting several cellular proteins, such as nuclear factor kappa-light-chain-enhancer of activated B cells (NF-κB), peroxisome proliferators-activated receptor-gamma (PPAR-γ) and its coactivator peroxisome proliferator-activated receptor gamma coactivator 1-alpha (PGC-1α), protein tyrosine phosphatase (PTP), forkhead transcriptional factors (the FoxO subgroup), adenosine monophosphate activated protein kinase (AMPK), CRE-binding protein regulated transcription coactivator 2 (CRTC2), endothelial nitric oxide synthase (eNOS), p53, myogenic differentiation (MyoD), liver X receptor (LXR), and transcription factor E2F1 (10, 11). Through its deacetylation activity, SIRT1 modulates functions of these critical molecules and shows its critical and multifaceted roles in cellular physiology (Figure 1).

Alterations of the level of SIRT1 expression were determined in several diseases including metabolic diseases, neurodegenerative diseases, cancer and aging. Whereas an increase in the expression of the SIRT1 protein was observed in cancer (12, 13), reductions in the SIRT1 level was more common in other diseases such as Alzheimer's Diseases (AD), Parkinson Disease (PD), obesity, diabetes, and cardiovascular diseases (14–18). Recent developments elucidated the relation between downregulation of SIRT1 levels and disease progression as an increase in the oxidative stress and inflammation (16, 17). For example, due to a significant decrease in SIRT1 levels which correlated with an increase in the oxidative stress parameters, accumulation of Tau proteins in AD, enhancement of acetylated p53 expression levels in coronary artery disease and increase in the fatty acid oxidation in obesity were observed in the patients (15, 17, 19, 20).

Previous studies showed that SIRT1 overexpression significantly increased cell viability, decreased cell apoptosis and reduced the release of pro-inflammatory cytokines (21–24). In addition, the regulation of metabolism and longevity by SIRT1 occurs through controlling the maturation of hypothalamic peptide hormones (25, 26). Specificity for SIRT1 increases in the relevant metabolic pathways in the hypothalamic circuitries which is also associated with altered downstream factors of SIRT1 such as FoxO transcription factors (27, 28). In the light of this information, we reviewed recent findings related to the association of the increasing level of SIRT1 protein rather than reduction of the SIRT1 expression and regulation of some disease related conditions such as obesity, cardiovascular diseases and neurodegeneration. The overarching aim of this paper is to provide a basis for hypothesizing that the level of SIRT1 are mechanistically increased to overcome the dysfunction of SIRT1 activity in the diseased conditions.

## SIRT1 and metabolic diseases

SIRT1 protein protects the functions of adipose tissue and liver in several aspects (29, 30) such as glucose homeostasis and fat metabolism against severe obesity (31, 32). It is also involved in energy balance and stress. Insulin sensitivity is increased in the pancreatic beta cells which have insulin resistance due to overexpression of SIRT1 (30, 33). The activity of PPARγ which have a role in the storage of glucose and fatty acid in adipose tissue is repressed by SIRT1 (34). During short term fasting, the CRTC2 is also depressed by SIRT1 and thus gluconeogenesis is declined in the liver tissue. During long term fasting, SIRT1 expression deacetylates and activates the PGC-1α to decrease adiposity and lipogenesis and to increase fatty acid oxidation (35–37). In addition, SIRT1 deacetlylates sterol regulatory element binding protein (SREBP), farnesoid X receptor (FXR), as well as liver X receptor (LXR) to increase bile acid production and to reverse cholesterol transport (30, 38). Thus, SIRT1 can be called as a “Master Metabolic Regulator” (30). Indeed, the dysregulation of energy sensing may cause inflammation and insulin resistance. Because of prevention of pro-inflammatory responses, SIRT1 behaves as a positive regulator of insulin in the adipose tissue (39). In one of the recent study, after feeding with high dietary fructose, the liver of rats were investigated in response to SIRT1 expression as a main energy sensing protein (40). However, they demonstrated a significant increase in the SIRT1 expression in the fructose-induced inflammation suggesting compensatory rise in the level of SIRT1 to decline the inflammation-related metabolic reactions (40). In addition, overexpression of SIRT1 in obesity which was formed by high-fat diet protects lipid-induced inflammation and hepatic steatosis while providing better glucose tolerance (41). These favorable effects of SIRT1 may be related with the activation of the antioxidant enzymes and stimulation of PGC1α to decrease the level of pro-inflammatory cytokines (41).

In some neurodegenerative diseases, a number of neuropeptide systems in the hypothalamus are affected from activity of SIRT1 which indicate an impact on metabolism. For example, SIRT1 upregulates the level of orexin receptor specifically in the lateral hypothalamic area and the ventromedial nucleus of the hypothalamus, whereas the expression of orexin and melanin-concentrating hormone is reduced in the hypothalamus due to inhibition of the active state of orexin neurons (25, 42, 43). In addition, SIRT1 also regulates the expression of BDNF in the brain. It was found that increased SIRT1 level diminished BDNF signaling which resulted in severe hyperphagia and obesity both in humans and animals (44, 45). These results showed that compensatory increase in the SIRT1 level to cope with the disease outcomes such as oxidative stress brings some additional metabolic dysfunctions in the body due to altered peptides in the endocrine system.

In addition to the obesity, SIRT1 has a role in the hepatic energy metabolism by modulating it nutritionally and hormonally. This modulation is mostly occurred through the deacetylation of metabolic regulators (46). Previous studies also showed that obese patients with non-alcoholic fatty-liver disease (NAFLD), which is the most common liver disease caused by elevated hepatic lipids, inflammation and oxidative stress, had high plasma levels of SIRT1 producing a potential against the physiological mechanisms related to NAFLD (47). In this type of disease, the action mechanism of SIRT1 acted through the modulation of PPARα activity and fatty acid oxidation (48).

On the other hand, it was found that increase in the SIRT1 activity upregulates genes-related metabolic functions, promotes insulin sensitivity and reduces inflammatory gene expressions in the adipose tissue of diet-induced obese animals (49). In addition, we found a polymorphism in the promoter region of *SIRT1* gene in obese children drawing attention to the association between altered SIRT1 activity and the risk of obesity (50). Previous reports also showed the protective role of SIRT1 on the development of osteoarthritis by upregulation of cartilage extracellular matrix genes and downregulation of matrix-degrading enzymes (51, 52). In addition, increase in the SIRT1 activity had a protective effect against osteoarthritis in animal models (53, 54). Therefore, the investigators suggested that the increase both in the activity and expression of SIRT1 might be a protective strategy for progression of osteoarthritis via the modulation of the NF-κB pathway (55).

## SIRT1 and age-related neurodegenerative diseases

The relationships between SIRT1 and age were investigated in the previous studies related to interaction of lifespan elongation and calorie restriction which is thought as an enhancer for SIRT1 activity (56, 57). Most of these studies showed that calorie intake restriction innervates the extension of life by the inducement of defense of cells against to free radicals and toxins for attenuation of apoptosis or amelioration of cell repair which are desired factors in aging (58–60). That means, altered SIRT1 expression and activity is thought to be a potent way to keep the cells and organs properly functioning for longer times. The positive correlation between age and SIRT1 expression and/or activity may be a compensation against unexpected situations such as oxidative stress (61, 62). For example, in one of our previous study, it was noted higher level of SIRT1 protein in older people compared with the SIRT1 level in the younger people (62). It was thought that increased protein level of SIRT1 in older people may be a compensatory mechanism due to accumulation of oxidative stress-related products and elimination of antioxidant enzyme level in elderly (62). However, increase in the expression does not mean increase in the activity of protein. An oxidative stress-dependent decrease in the SIRT1 activity was noted in aged animals that had high levels of SIRT1 protein (63, 64). The decline in the activity of SIRT1 may not be related directly with SIRT1 protein but also its downstream or upstream molecules such as a decline in NAD+ levels with aging (65).

One of the main risk factor for several neurodegenerative diseases such as Alzheimer's disease (AD) and Parkinson's disease (PD) is age. The common underlying mechanisms of neurodegeneration are increase in the neuroinflammation, mitochondrial damages and oxidative stress (66, 67). In literature, it was shown that sirtuins' hyperactivity could reduce these negative outcomes both *in vivo* and *in vitro* due to its neuroprotective role (68–71). In the AD pathology, SIRT1 deacetylates substrates in favor of the non-amyloidogenic pathway or acts directly on the Aβ and Tau proteins (72). Molecular studies showed that SIRT1 activation prevents the accumulation of Aβ plaques and tau pathology through the NF-κB signaling pathway by upregulation of the *ADAM10* gene, induction of the Notch pathway, and inhibition of the mTOR pathway (20, 73, 74). As shown in previous studies, SIRT1 epigenetically reprograms inflammation taking about AD formation at the earlier stages by altering transcription factors (24, 75, 76). In addition, it was observed that brains of AD patients have consistently reduced NAD+ levels and SIRT1 transcription and/or protein levels involved in chronic inflammation that can also be altered by increased levels of the activated proinflammatory transcription factor NF-κB (77–79). In one of our studies, we found a significant increase in the SIRT1 level of dementia patients (80). Furthermore, in the patients with Huntington's disease (HD), Baldo and his colleagues found higher expression of SIRT1 protein level in the most affected brain regions, especially hypothalamic regions important for metabolic regulation, compared to brain regions which were less affected from the mutant huntingtin protein (28). In PD, SIRT1 inhibits α-synuclein aggregation by deacetylating proteins such as heat shock proteins and PGC-1α and, therefore, it protects dopaminergic neurons against cell death which occur due to the formation of insoluble fibrils called Lewy bodies (81, 82). In the *in vitro* PD model, it was observed that an overexpression of SIRT1 due to application of toxin (rotenone or MPTP) which causes neurodegeneration was rescued cells from oxidative stress (16, 83). The neuroprotection against to PD occurred by the mechanism of decreasing in the expression of NF-κB and cleaved PARP-1. In a postmortem study, the levels of SIRT1 showed a slight increase in the dementia patients with Lewy bodies (16, 83). However, as seen in the AD, the activity of the SIRT1 protein also decreased in the PD patients producing neurodegeneration in correlation with possible higher oxidative stress, synaptic and cell loss, and neuroinflammation (16).

We thought that the high levels of SIRT1 protein might have a role in alleviating the oxidative stress that is significantly increased in neurodegeneration because an induction of the SIRT1 expression occurs when the organism encounters a biological stress such as aging or an age-related disorder due to the role function of SIRT1 as an important stress sensor molecule.

Also, in literature, it was stated that high levels of SIRT1 may increase the expression of genes related to neuronal protection (84–86). On the other hand, SIRT1 behaves as a double edged sword in response to inflammation which is a cause of neurodegeneration. That means, low levels of SIRT1 cause early acute inflammation-related damages to tissues by increasing NF-kB, and high levels of SIRT1 during late inflammation cause immunosuppression and increased the rate of death (87). In a previous study, investigators observed that increase in the expression level of SIRT1 cannot protect the brain from neurodegeneration without increasing the activity level of SIRT1. For example, Ciriello and his colleagues observed a significant decrease in the level of phosphorylated SIRT1, the active form of SIRT1, in the patients with multiple sclerosis (88). Interestingly, a significant negative correlation between phosphorylated and non-phosphorylated forms of SIRT1 was observed explaining both the SIRT1 overexpression and inactivity of SIRT1 in diseased state. In addition, when a SIRT1-activating molecules were given to the organisms, profound therapeutic benefits and neuroprotective effects were recorded against age-dependent neurodegenerative diseases (89).

## SIRT1 and cardiovascular disease

Nowadays, the sirtuin protein family is thought as one of the important target for cardiovascular diseases (CVD). Therefore, the role of SIRT1 protein and its downstream molecules also gains importance in the experimental studies related with CVD development. In cardiomyocytes, during prenatal period, SIRT1 is found in the nucleus, however, it is mostly located in the cytoplasm of myocytes of adult heart of rodents (90). Previously, we found that the level of the SIRT1 expression was significantly higher in the CVD patients compared to the levels of SIRT1 in healthy subjects pointing the cross-talk between SIRT1 protein expression and reactive oxygen species (61). In this previous study, we also found a significant increase in the oxidative stress parameters which may be an inducer for SIRT1 expression. In cardiomyocytes, myoblast gains resistance against to oxidative stress by increasing expression of nuclear SIRT1 protein. To do produce this antioxidative activity, SIRT1 protein enhances the level of MnSOD expression through p53 deacetylation (90). In addition, activation of FoxO1-dependent oxidative pathway by overexpression of SIRT1 protein is another regulatory way of protection of cardiomyocytes from oxidative stress (91). By the help of this pathway, cardiac infarct volume is reduced to ameliorate and recover cardiac function after ischemia/reperfusion in mice (92). It is also thought that transcriptional activity of NF-κB protein, a preconditioner in cardiac ischemia, is inhibited by SIRT1 protein to promote cell protection (93, 94). In literature, it was reported that the activity of SIRT1 protein is directly or indirectly controlled via the JNK1-SIRT1 link by accumulated oxidative stress which is caused by an increase in the ROS level due to aging or age-related diseases enzyme (1). It was demonstrated that ROS inhibited JNK phosphatases which activated the JNK1 to phosphorylate SIRT1 (95, 96). Furthermore, this phosphorylation increased the activity of SIRT1 resulting its translocation into the nuclei (97). Alcendor et al. (91) noted that the rate of SIRT1 overexpression had two-sided action in the cardiovascular system. For example, 2.5- to 7.5-fold increase in the SIRT1 expression attenuated apoptosis, the symptoms of cardiac dysfunction, age-related cardiac hypertrophy and expression of senescence markers. On the other side, 12.5-fold increase in the expression of SIRT1 resulted in increased cardiac hypertrophy due to oxidative stress and apoptosis. This study explained clearly the relation between oxidative stress and overexpression of SIRT1 to the pathological levels in CVD patients. In one of our previous studies (61), we found a positive correlation between total antioxidant level and SIRT1 level in CVD patients. Therefore, we can conclude that the increase in the SIRT1 level may be a compensatory mechanism to increase the antioxidants against oxidative stress in CVD patients.

Contrary to some previous studies (98, 99), the SIRT1 level significantly decreased approaching to control values in the CVD patients receiving statin therapy (100). The decline in the SIRT1 level by statins can be explained by the statins' inducement effect on PPARγ activity to protect patients against the progression of atherosclerosis (101). On the other hand, PPARγ inhibits SIRT1 expression at the transcriptional level which interrupting compensatory action of increased SIRT1 expression (102).

## Conclusion

Recent studies have shown that age-related diseases or endocrine system dysfunctions are associated with an increase in SIRT1 expression levels, but with a decrease in their activity. The oxidative stress produced during these processes may lead to compensatory or protective increase in the SIRT1 expression to deal with the decline of the SIRT1 activity.

## Author contributions

BE wrote the draft of the manuscript and UK finalized the manuscript.

### Conflict of interest statement

The authors declare that the research was conducted in the absence of any commercial or financial relationships that could be construed as a potential conflict of interest.
